# Parental Perspectives on Eating Disorders of Their School-Age Children with ADHD in Hong Kong: A Qualitative Study

**DOI:** 10.3390/nu17030513

**Published:** 2025-01-30

**Authors:** Shu-Cheng Chen, To Ming Stanley Wu, Han Li, Jia-Wen Shou, Jing Qin, Guo-Tao Wu, Wai-Yin Cheng, Wing-Fai Yeung

**Affiliations:** 1 Centre for Smart Health, School of Nursing, The Hong Kong Polytechnic University, Hung Hom, Hong Kong, China; shucheng.chen@connect.polyu.hk (S.-C.C.);; 2 Department of Rehabilitation Sciences, The Hong Kong Polytechnic University, Hong Kong, China; 3 School of Nursing, The Hong Kong Polytechnic University, Hong Kong, China; 4 Li Dak Sum Yip Yio Chin R&D Centre for Chinese Medicine, The Chinese University of Hong Kong, Hong Kong, China; 5 Psychology Department, Southwest University, Chongqing 400715, China; 6 Department of Food Science and Nutrition, The Hong Kong Polytechnic University, Hong Kong, China; 7 Research Center for Chinese Medicine Innovation, The Hong Kong Polytechnic University, Hong Kong, China; 8 Research Institute for Smart Ageing, The Hong Kong Polytechnic University, Hong Kong, China

**Keywords:** ADHD, eating problems, parental strategies, qualitative research, school-age children, Hong Kong

## Abstract

**Background:** Children with attention deficit hyperactivity disorder (ADHD) frequently encounter eating problems. However, qualitative research on the eating problems of these children and the strategies employed by their parents to manage these issues remains limited. This study aimed to explore the eating problems of school-age children with ADHD and the coping strategies used by parents in urban settings like Hong Kong. **Methods:** A descriptive qualitative design was utilized, employing semi-structured focus group interviews. Purposive sampling was used to recruit 12 parents who voluntarily participated in five focus group sessions. The interviews were conducted in Cantonese, audio-recorded, and transcribed verbatim. Data were analyzed using template thematic analysis to identify key themes and subthemes. **Results:** Two major themes emerged: challenges affecting ADHD children’s eating behaviors and parental coping strategies in Hong Kong. Children’s eating difficulties stemmed from ADHD-specific behaviors, compounded by Hong Kong’s demanding educational system and urban environmental constraints. In response, parents developed multifaceted coping approaches, ranging from dietary modifications and behavioral management strategies to healthcare resource utilization, while adapting their urban lifestyle to accommodate their children’s needs. **Conclusions:** Children with ADHD face eating challenges that intersect with Hong Kong’s sociocultural environment, where educational pressure, limited living spaces, and parents’ work schedules influence their eating patterns. Parents adopt integrated Eastern–Western approaches, supported by Hong Kong’s comprehensive healthcare resources spanning professional networks and community programs. Evidence-based dietary guidelines are essential to address ADHD-related nutritional misconceptions.

## 1. Background

### 1.1. Attention Deficit Hyperactivity Disorder (ADHD)

ADHD is a neurodevelopmental disorder characterized by persistent patterns of inattention, hyperactivity, and impulsivity that interfere with functioning or development [1]. It is one of the most common mental disorders in children, with an estimated incidence rate of 5–8% globally [2,3,4]. The primary symptoms of ADHD include difficulty sustaining attention, excessive activity, and impulsive behavior [5]. ADHD frequently coexists with other psychiatric and developmental disorders, such as anxiety, depression, and learning disabilities, complicating its clinical presentation and management [6]. This disorder considerably influences not only the affected children, leading to challenges in academic performance and social relationships, but also their families, who often experience heightened stress and disruption in daily routines [7]. Conventional interventions for ADHD typically involve pharmacological treatments, such as stimulant medications, and behavioral therapies [8]. However, these interventions have limitations, including side effects, variability in individual response, and insufficient long-term efficacy in some cases [9]. 

### 1.2. Eating Problems of Children with ADHD

Children with ADHD frequently encounter considerable eating problems, which can present as irregular eating patterns, diminished appetite and overeating, and a tendency towards unhealthy food choices [10,11]. These patterns vary significantly among ADHD subtypes, with predominantly inattentive and combined types showing higher risk of overweight status, while the hyperactive type is often associated with a lower BMI [12]. For example, among children with ADHD, 29% were at risk of being overweight and 17.3% were classified as overweight [11], with a higher prevalence observed in the inattentive and combined subtypes [13]. These issues often include symptoms such as skipping meals, overeating, food selectivity, and a preference for high-sugar and high-fat foods [14,15]. Such eating problems can negatively affect physical health, leading to obesity, nutritional deficiencies, and poor growth patterns [16,17]. In school-age children, these eating problems can exacerbate ADHD symptoms, adversely affecting their cognitive function, energy levels, and overall academic performance [18,19]. The causes of eating problems in children with ADHD are multifaceted, involving a combination of genetic, neurobiological, and environmental factors [20]. Neurobiological factors include the dysregulation of dopamine pathways, which play a critical role in reward processing and appetite control [21]. The impulsivity and inattention characteristic of ADHD can hinder the development of healthy eating habits [22], particularly affecting those with the predominantly inattentive type through a decreased awareness of satiety cues [23]. ADHD medications, particularly stimulants, can contribute to eating problems by suppressing appetite, leading to reduced food intake and weight loss [24,25]. Without medication, children may demonstrate increased impulsive eating behaviors and difficulty in recognizing satiety signals [26]. Environmental influences, such as family mealtime dynamics and the availability of healthy food options, play a crucial role [27,28]. Addressing these eating problems is essential for improving the overall health and well-being of children with ADHD, necessitating a multidisciplinary approach that encompasses dietary management [29], behavioral strategies [30], and parental training and education [31,32].

### 1.3. Research Gap

Eating problems among children with ADHD are prevalent, and the effect these issues have on their health and development is considerable. However, most existing studies focus predominantly on the core and behavioral symptoms of ADHD, often overlooking the nuanced dietary challenges and parental feeding practices. A notable gap exists in the literature regarding the specific eating problems of school-age children with ADHD and the strategies employed by parents to manage these problems [10,18]. While some research has recognized the influence of ADHD interventions on appetite and eating patterns [24,33], qualitative data capturing the firsthand experiences and perspectives of children and their parents in real-world settings are limited. This gap is particularly pronounced in the context of densely populated urban environments, such as Hong Kong, where unique cultural, social, and environmental factors may influence dietary habits and parental strategies [34]. The present study aimed to address this gap by utilizing focus group interviews conducted in Hong Kong to delve into the eating habits of school-age children with ADHD, the specific dietary issues they face, and the coping strategies parents employ. The results could provide valuable insights into the multifaceted nature of eating problems in children with ADHD within a supercity context and inform more effective, tailored support to these families.

## 2. Methods

This study utilized a descriptive qualitative design [35], grounded in naturalistic inquiry, to achieve a comprehensive understanding of a specific event. The research findings were reported in alignment with the Consolidated Criteria for Reporting Qualitative Research [36]. 

### 2.1. Participants and Settings

Recruitment occurred in Hong Kong between February and March 2024, utilizing promotional posters on Facebook and invitations via WhatsApp (Meta Platforms, Inc., iOS Version 24.2.1). Parents of school-age children with ADHD were invited to participate. The participants joined the individual interviews to discuss their experiences and perspectives on their children’s eating behavior and parental feeding strategies. Written information about the qualitative study was provided to potential participants to assess their interest in participation. The informed consent form comprehensively detailed the study’s purpose, design, methodology, benefits, risks, and data privacy protocols and included contact information for further inquiries. The inclusion criteria specified that participants must be (1) parents of children aged 6–8 years with a documented ADHD diagnosis and (2) residents of Hong Kong who were fluent in Cantonese. The participants’ preferred interview times were recorded, and mutually convenient schedules were arranged. Following a thorough explanation of the study’s purpose, procedures, and importance, the participants provided a written informed consent. The participants were also informed about the use of audio recordings, assured of the confidentiality of their information, and apprised of their right to withdraw from the study at any time without penalty. Focus group interviews, a qualitative research method that gathers data through guided small-group discussions, were conducted on the campus of The Hong Kong Polytechnic University. This method was chosen because it enables researchers to collect rich data through participant interactions and group discussions. A sample size of approximately 12–24 participants was determined necessary to achieve thematic saturation—the point at which additional interviews no longer generate new themes or substantial insights about the research topic [37]. Thematic saturation helps ensure that sufficient data has been collected to comprehensively understand the phenomenon being studied. Purposive sampling was selected as the most appropriate approach for this study for several reasons. First, it allowed us to identify participants who could provide rich, detailed information about the specific feeding experiences of children with ADHD in Hong Kong. Second, this sampling method enabled us to efficiently recruit participants that met our precise inclusion criteria while managing resource constraints. To ensure participant relevance and minimize selection bias, we implemented a structured screening process requiring (1) a documented ADHD diagnosis from qualified healthcare providers, (2) verification of child’s age within the specified range, and (3) confirmation of the parents’ direct involvement in daily feeding activities. Participant selection was conducted by two researchers (SCC and HL) using these predetermined criteria to minimize subjective judgment. 

### 2.2. Data Collection 

The interview guide, comprising seven open-ended questions in Chinese (Table 1), was initially developed by the first author (SCC). This guide was subsequently revised by an experienced ADHD researcher (GTW) and a qualitative research expert (WFY). Semi-structured focus group interviews were conducted face-to-face by two moderators (SCC and LYP). While traditional focus groups often include 6–8 participants, we deliberately chose smaller groups of 2–3 participants for 3 specific reasons. First, given that ADHD has genetic components, some participating parents may also experience attention-related challenges, making smaller groups more conducive to sustained engagement. Second, the intimate setting of 2–3 participants provided a more confidential environment, particularly important given the stigma often associated with ADHD diagnoses. Third, this format enabled a more in-depth exploration of individual experiences and allowed each participant adequate time to fully articulate their thoughts about the learning platform. A pilot test was conducted with two participants from the first interview group to ensure the comprehensibility of all questions. All interviews were conducted in Cantonese. At the commencement of each session, the moderators introduced themselves, outlined the purpose and procedures of the interview, emphasized the importance of confidentiality, and addressed any questions from the participants. Data collection was halted when thematic saturation was achieved. All interviews were audio-recorded and transcribed verbatim in Chinese prior to data analysis. Participants were offered a cash incentive of HKD 200.

### 2.3. Data Analysis

All interviews were recorded and transcribed verbatim in traditional Chinese prior to data analysis. Each individual was assigned a unique, randomly generated code to ensure participant confidentiality. Descriptive characteristics were employed to summarize the demographic data. Data analysis was conducted using template thematic analysis [38], which is a systematic method for identifying patterns and themes within qualitative data. This process involved developing a thematic code tree—a hierarchical structure that organizes the identified themes from broad categories to specific subthemes. Key quotations, which are representative verbatim excerpts from participants’ responses, were extracted from the transcripts to illustrate and support each identified theme in our findings. Two researchers (SCC and HL) independently read each transcript multiple times to thoroughly familiarize themselves with the data, aiming to deeply understand the participants’ perspectives on their children’s eating habits and parental coping strategies. The researchers manually performed line-by-line coding, condensing the codes based on identified similarities and differences. Higher-level abstract codes were then extracted and organized into subthemes and main themes. Any disagreements between the researchers were resolved through discussion until consensus was reached. Finally, the identified themes and subthemes, along with their representative quotes, were translated into English.

### 2.4. Trustworthiness

Several procedures were implemented to ensure the trustworthiness of the qualitative data collected from focus groups on the basis of the criteria established by Lincoln: credibility, dependability, confirmability, and transferability [39,40]. For credibility, the focus group protocol was revised during two group meetings, and the discussion questions were pilot tested with two participants. Investigators were selected on the basis of their requisite knowledge and expertise in qualitative research, and purposive sampling techniques were employed to select focus group participants. Dependability was achieved through regular debriefing sessions with WFY, an expert in qualitative studies, and by providing participants with the findings, including the thematic code tree and key quotations, for comments and confirmation. The coding structure was validated by another researcher (GDW) and revalidated by the corresponding author (WFY) through a review of the original transcripts. Confirmability was ensured by returning transcripts to the participants for feedback, maintaining a critical and honest stance through self-scrutiny, and employing negative case analysis to enhance the validity of interpretations. Finally, transferability was addressed by applying the saturation theory to determine the richness of the data [41], with data collection continuing until new information was nearly exhausted by the third focus group session [42].

## 3. Results

The focus group interviews were conducted between March and April 2024. A total of 38 parents were invited to participate in the study, and 12 accepted the invitation. A total of five focus group interviews were conducted. The interviews had a mean duration of 53.4 min and ranged from 47 min to 59 min. The number of participants in each group ranged from two to three. The participants in each group were unfamiliar with one another. Data saturation was applied to guide the data collection [41], with themes and subthemes that were established in the third focus group interview session and enriched after the fifth focus group session.

### 3.1. Sample Profile

The twelve parents (ten females [83.3%] and two males [16.7%]) of children with an ADHD diagnosis attended the five sessions. The mean age of the parents was 40.1 years (SD = 3.7), and the mean age of their children was 6.9 years (SD = 0.9). The participants’ demographic information is presented in Table 2.

### 3.2. Major Themes

In terms of the parents’ expression of their children’s eating problems and their parental coping strategies, two main themes were identified from the data: (1) challenges affecting ADHD children’s eating behaviors in Hong Kong and (2) parents’ coping strategies in Hong Kong. The specific subthemes under each theme were described, and Table 3 presents the code structure. Following the established thematic analysis methodology [43], our analysis focused on identifying meaningful patterns across the dataset. Our interpretations were shaped by our theoretical frameworks and research context, acknowledging that different researchers might develop varying interpretations recognized strength in qualitative inquiry. Through team discussions and reflexive practice, we ensured our interpretations remained grounded in the data. In selecting illustrative quotes, we considered: (1) how the quote captured the participants’ lived experiences; (2) the richness and depth of expression in conveying the concept; and (3) how well it represented the essence of the identified theme. While we note when multiple participants (≥4) expressed similar views, the significance of the themes was determined by their capacity to illuminate meaningful aspects of the phenomenon under study rather than quantitative prevalence.

#### 3.2.1. Challenges Affecting ADHD Children’s Eating Behaviors in Hong Kong

The challenges presented three subthemes: (a) ADHD-specific eating behaviors, (b) challenges related to the local educational system, and (c) challenges related to the urban environment.

ADHD-Specific Eating Behaviors

The participants reported various challenges regarding their children’s eating problems, categorized into ADHD eating patterns, the impact of ADHD medication, and ADHD-related allergic reactions. The parents described mealtimes as particularly stressful when children exhibit impulsive and hyperactive behaviors; one parent noted, “Sometimes when his emotions flare up, he becomes very unreasonable. Mealtimes are always stressful. He can’t sit still, keeps moving around, and needs constant supervision.” (031). Another common issue was the erratic engagement with food, with children often leaving the table if not immediately interested in eating: “Most of the time, if he’s not really hungry, he’ll just walk away and do something else first.” (024). The impact of ADHD medication further complicated eating behaviors, as it tended to significantly reduce appetite and can also affect sleep, growth, and emotional stability: “He already eats very little, and after his ADHD medication, his appetite drops even more. He barely eats a few bites, and no matter what we offer, it seems useless.” (015); “ADHD medication affected his sleep, reduced his appetite, impacted his height growth, and also caused emotional problems.” (059). Additionally, ADHD-related allergies presented another layer of complexity, requiring parents to vigilantly monitor and adapt diets to manage sudden allergic reactions to common foods: “When he was young, he suddenly itched after eating salmon, then eggs… once he suddenly became allergic to beef, itching after eating it.” (031); “He has respiratory issues when eating citrus, oranges, strawberries, and mangoes, so we have to be careful with his diet.” (012).

Challenges related to the local educational system

Educational challenges were related to the school schedule, academic workload, and teacher–student interactions. School schedules in Hong Kong are particularly demanding, with early starts and long days that disrupt normal eating patterns, as one parent explained, “In the morning, I prepare breakfast for him, and even though he should be hungry, he often doesn’t eat—perhaps waking up early affects his appetite.” (001). The extended school hours also leave little time for other activities, further impacting children’s well-being: “Hong Kong students’ daily school schedules are really long—by the time they get home after school, there’s basically no time left for other activities.” (012). Additionally, the heavy academic workload exacerbates stress, with extensive homework that overwhelms these children and complicates time management: “In primary school, homework just piles up—that’s his focus. Everything is laid out there, making it difficult to manage time.” (043). Negative teacher–student interactions also profoundly affect these children, leading to emotional distress and decreased appetite: “He came home saying the teacher scolded him, which affected his mood completely. He barely had any appetite for dinner.” (001); “His teacher treats him as if he has intellectual disabilities… these students already struggle to cope with regular classes—if teachers are also impatient with them, they simply cannot survive in school.” (059).

Challenges related to the urban environment

This subtheme reflects key factors contributing to the difficulties of parents living in supercities like Hong Kong. Extended working hours for parents were a significant concern, with one parent noting, “There was a period when I was extremely busy and couldn’t take care of him. Both his father and I leave early and return late from work.” (001), indicating the limited parental supervision available due to job commitments. This often necessitates reliance on domestic helpers, which introduces its own set of challenges: “I believe healthy eating habits start with regular mealtimes and appropriate portions. Since I have to work, I rely on our domestic helper to take care of him. The domestic helper has limited authority—when she’s watching the children, they will read while eating because they don’t respect her.” (031), highlighting issues of authority and adherence to structured eating practices. Additionally, the ubiquity of fast-food options poses a further obstacle, particularly in densely populated urban areas where convenience often overrides nutritional consideration: “When we lived in Ap Lei Chau, there was a McDonald’s nearby. He would always insist on having the McChicken meal and refuse to eat anything else. He absolutely had to have McDonald’s.” (012), illustrating the temptation and ease of access to less healthy food choices in Hong Kong’s compact residential districts. Cramped living conditions also exacerbate these challenges, as limited space can disrupt mealtime routines and behavior management: “When preparing dinner, because our home is small, we all eat together in the living room where space is limited. When he can’t sit still and moves around, there’s not much we can do.” (024).

#### 3.2.2. Parents’ Coping Strategies in Hong Kong

Parents’ strategies revealed four subthemes: (a) ADHD dietary strategies, (b) behavioral management, (c) Hong Kong healthcare resource utilization, and (d) urban lifestyle adaptation.

ADHD Dietary Strategies

Dietary adjustments have been adopted on several aspects. One approach was avoiding foods with artificial additives believed to affect emotional stability: “I know that foods with too many artificial colorings can affect emotional stability, so now I pay special attention to choosing healthy foods for my children and won’t let them eat carelessly.” (012). A parent eliminated white rice and sugar, substituting them with alternatives like cane or brown sugar to mitigate hyperactivity: “I heard that white rice isn’t good for ADHD children, so our whole family stopped eating white rice… we also stopped using white sugar, switching to cane sugar or brown sugar because white sugar has many chemicals that make ADHD children more hyper.” (043). Another said: “I mainly buy avocado, nuts, fish… telling him not to eat so many snacks, I saw a news report saying they affect attention.” (013). Additionally, probiotic-rich foods were incorporated based on their potential benefits for gut health, which was linked to cognitive and emotional well-being: “After hearing a university study that certain probiotics are good for children’s intestines, the body’s second brain, I try to find these foods for him, like yogurt or Yakult.” (012). Traditional Chinese dietary methods were also utilized, one said: “Because I have seen a Chinese medicine practitioner frequently sharing on social media about how to strengthen the spleen, discussing purple yam powder and black bean powder, and I prepare these drinks for my son.” (052).

Behavioral Management

The parents employed several behavioral management strategies to improve eating habits, focusing on physical activity, innovative mealtime tactics, and emotional regulation. Physical exercises like orienteering are used to stimulate appetite, as one parent notes: “Inducing hunger makes him willing to eat… On Fridays he participated in orienteering, running for two hours, then going to school and coming back, he would actually ask for food” (012). Innovative eating elements include incentives and clever tableware adjustments; for example, parents use time-bound rewards to motivate their children: “Using rewards, for example, I tell my son if he eats earlier, then Dad will play with him for a while… if he finishes eating by 9, he’ll have 15 min to play; if he’s late, the playtime will be reduced.” (004), and oversized bowls to visually reduce portion sizes: “My friend’s child eats very little, so he humorously bought a large bowl to make the usual amount look small, making the child think they finished it in just two bites.” (050). Involvement in food preparation also encourages eating: “She buys groceries himself or helps with cooking, and she eats more…” (015). Regulating emotions through enjoyable and timely meals is crucial; one parent describes how improving the mealtime environment positively impacted their child’s appetite: “Because he has emotional issues, I put effort into making things he likes and make fun shapes in his lunchbox… Now he’s in second grade, he has become quite chubby… Since he gets very irritable when hungry, dinner must always be on time.” (031).

Hong Kong Healthcare Resource Utilization

The parents utilized a blend of healthcare resources in Hong Kong. Some parents explored non-pharmaceutical treatments to promote healthy development: “We’re currently considering exercise therapy or acupuncture as treatment options for my child.” (012); “I’ve tried non-pharmaceutical treatments on my child, such as acupuncture and *tuina*, to support their healthy development.” (001). Health information resources also play a critical role, with parents frequently consulting medical columns in magazines and attending seminars to gain insights into ADHD management: “We read medical columns in magazines and newspapers where doctors share advice on what soups to drink and what foods can help boost a child’s health and reduce frailty.” (043); “I attended a talk by a psychiatrist at Heep Hong Society who mentioned that ADHD medications can reduce children’s appetite, and long-term use of ADHD medications might lead to high blood pressure, high blood sugar, and high cholesterol.” (012). Professional support networks are crucial, with parents engaging in university research and open courses that provide training and resources: “We joined a university study using pediatric *tuina* for ADHD. The practitioners analyzed the issues and taught us the techniques… They even sent me teaching videos afterward—it was excellent.” (013); “Over these past few years, we’ve been regularly taking our son to various classes and different centers, and he has also participated in social skills groups.” (004). Peer support among parents is another valuable resource, where sharing experiences and tips online helps in discovering new coping strategies: “I look up other ADHD mothers’ suggestions online. For example, someone mentioned cooking partridge soup with Tiger’s Milk mushroom and *Lingzhi*” (050); “I often read friends’ shared experiences and online information, which consistently mention that ADHD medications affect appetite, so I keep reminding myself about this.” (013). Lastly, school–family collaboration is emphasized to ensure a supportive educational environment without over-reliance on medication: “If he’s not disrupting classroom order, there’s no need for medication. Even now that he’s in Primary 1 and 2, I regularly discuss with his class teachers.” (012).

Urban Lifestyle Adaptation

Urban lifestyle adaptation plays a crucial role for ADHD families in Hong Kong. Parents adjusted their family routines to accommodate the needs of their children. A mother mentioned: “Lack of sleep affects his mood and eating habits… so we’ve adjusted to having dinner at 6 p.m. and bedtime at 8:30 p.m., allowing proper digestion before sleep.” (031). Adjustments to the home environment are also significant. Some families preferred to limit the influence of extended family members, like grandparents, to maintain consistent discipline and routines. One said: “it’s better that we don’t live with the grandparents normally, as there’s less intervention. They tend to be too permissive with their grandchild…” (012). Additionally, eating environment adjustments were implemented to improve focus and eating habits: “During his toddler years, he needed TV while eating. We transitioned from TV to children’s songs, and finally to picture books during meals.”, a parent mentioned (050). The balance between home-cooked and outside meals was carefully managed: “I think environmental factors are very important—eating out versus eating at home is different. When we’re out, I allow them to eat less healthy foods… But at home, it must be healthy—home-cooked meals are never deep-fried, and we use less sugar and salt.” (052). Furthermore, the integration of domestic helpers into the family’s routine involved specific instructions to ensure that dietary guidelines are followed, enhancing consistency in the child’s diet: “I remind our domestic helper how to feed my children, for example, serving the child enough portions of rice…” (031). 

## 4. Discussion

### 4.1. Main Findings

This study aimed to explore the eating problems of school-age children diagnosed with ADHD and the coping strategies employed by their parents in urban areas like Hong Kong. The use of descriptive semi-structured, face-to-face focus group interviews facilitated rich, dynamic discussions that uncovered a wide range of parental strategies and challenges, enhancing the depth of the data collected [44]. This study is the first to specifically focus on the eating problems of school-age children with ADHD and the real-world coping strategies of their parents within the context of a developed and densely populated urban environment like Hong Kong. This unique cultural, social, and environmental context provided a rich backdrop for understanding the dynamics of eating problems in children with ADHD. The findings contribute valuable insights that could inform more effective, tailored interventions to support families dealing with ADHD in similar urban settings. Through a thematic analysis, two major themes emerged: (1) challenges affecting ADHD children’s eating behaviors in Hong Kong and (2) parents’ coping strategies in Hong Kong. The key findings revealed the multifaceted nature of eating challenges among children with ADHD, encompassing ADHD-specific eating problems, as well as Hong Kong’s educational and environmental challenges. Furthermore, parents employed a range of strategies to manage their children’s eating problems, including ADHD dietary approaches, behavioral management, Hong Kong healthcare resource utilization, and urban lifestyle adaption.

### 4.2. Comparison with Previous Studies

The findings of this study align with several observations from earlier research. First, the difficulties related to children’s eating problems, such as slow eating, distraction, and picky eating, match those observed in earlier studies on children with ADHD [10]. These problems can exacerbate mealtime challenges and increase parental stress, as noted in previous literature. Second, the influence of ADHD symptoms on eating problems, such as hyperactivity and inattention, is in accord with recent studies that highlighted the role of neurobiological factors in disrupting regular eating patterns [45]. Lastly, the negative effect of ADHD medication on appetite and eating problems, such as reduced food intake and weight loss, has been consistently documented in prior studies [46,47,48,49,50]. The present study also highlighted considerable issues related to children’s constitution, such as allergies and poor gastrointestinal function, which affect their eating problems. These findings suggest that the physiological challenges faced by children with ADHD are compounded by their eating issues, necessitating a comprehensive approach to dietary management that considers behavioral and medical factors. The literature indicates that children with ADHD often have comorbid conditions like gastrointestinal issues and food allergies, which can further complicate their eating problems [51,52]. The considerable effect of allergies and gastrointestinal issues on children’s eating problems warrants further investigation. Our findings reveal the co-occurrence of allergic conditions among participants with ADHD, which aligns with growing evidence of this association in the literature. Recent meta-analyses have shown that individuals with ADHD have a significantly higher prevalence of allergic diseases compared to those without ADHD [53]. The biological mechanisms underlying this association may involve shared inflammatory pathways and neuroimmune interactions. Studies suggest that inflammatory mediators can affect dopamine and norepinephrine systems, which are implicated in ADHD pathophysiology [54]. Additionally, both conditions share common genetic risk factors and environmental triggers that may contribute to their co-occurrence [55]. This overlap highlights the importance of considering allergic conditions in ADHD clinical management and suggests potential shared pathophysiological mechanisms that warrant further investigation.

However, notable inconsistencies can be observed between the findings of the present study and those of earlier studies. Contrary to some research that suggests a minimal effect of family mealtime dynamics on children’s eating behaviors [56], the results of the present study highlight considerable family conflicts and psychological stress associated with managing these eating problems. The possible explanation for this discrepancy may be the unique sociocultural context of Hong Kong, where family cohesion and parental expectations are particularly emphasized, potentially intensifying the stress experienced by parents [57]. Additionally, the narrow living spaces in Hong Kong [58], coupled with immense living pressures, may contribute to heightened family tensions and conflicts during mealtimes. While some studies have reported that educational interventions alone are sufficient to manage eating problems in children with ADHD [59], the findings of the present study suggest that a multifaceted approach, including dietary adjustments, environmental modifications, and physical activity, is more effective. The development of the city likely makes parents more focused on education and more patient with their children [60]. They have more channels through which to learn scientific methods for managing ADHD and access to more resources than those in less developed areas [61]. This increased access to information and resources enables parents to implement a broader range of strategies to effectively manage their children’s eating problems. Additionally, while participants demonstrated BMI values within the normal range, parental reports indicated concerns about lean body composition and slower weight gain. This pattern aligns with the existing literature documenting differential weight presentations across ADHD subtypes. Previous research has established that inattentive and combined subtypes tend toward a higher BMI [12], while predominantly hyperactive presentations often associate with normal to lower BMI ranges. In our interviews, parental concerns primarily centered on selective eating, nutritional adequacy, and maintaining adequate weight, rather than weight excess. This pattern may reflect several factors: first, our sample potentially included a higher proportion of hyperactive-type ADHD children; second, the low prevalence of medication use among participants may have influenced the observed eating behavior patterns; and third, the unique dietary culture and lifestyle patterns in Hong Kong may have contributed to these outcomes. These findings suggest the need for future research to systematically document and analyze ADHD subtype distribution, explore dietary behavioral variations across subtypes, and examine the impact of medication status on eating behaviors.

Some parents reported using innovative methods to encourage their children to eat, including using large bowls to create the illusion of smaller portions and using food coloring to make meals more appealing. These strategies highlight the parents’ willingness to experiment with unconventional methods to overcome eating challenges. Previous research supports the idea that visual and sensory enhancements can improve children’s eating behavior by making food more attractive and engaging [62]. Involving children in food preparation emerged as another effective strategy, with parents noting increased food intake when children participated in buying groceries or cooking. This approach fosters improved eating habits and a sense of responsibility and engagement, aligning with the concept of participatory education. Studies have shown that involving children in food preparation can increase their willingness to try new foods and improve their overall dietary habits [63,64]. The innovative eating methods suggest a need to explore creative, child-friendly strategies for managing eating problems in children with ADHD. Future studies could investigate the effects of playful techniques and the involvement of children in food preparation to managing ADHD-related eating problems.

Notably, Hong Kong possesses comprehensive healthcare resources and support systems, including professional networks, university research programs, and community health organizations that can be leveraged to develop and disseminate evidence-based dietary guidance, providing a foundation for implementing targeted nutritional education programs for ADHD families. However, the analysis of parental narratives revealed prevalent misconceptions regarding the dietary management of ADHD. Parents reported various dietary modifications and restrictions based on common beliefs about food impacts on ADHD symptoms. Many of these dietary interventions lack evidence support; particularly, the widely held belief about sugar inducing hyperactivity has been consistently disproven in controlled studies [65,66]. While elimination diets show limited efficacy in specific cases of food sensitivities, general food category restrictions remain unsupported by current evidence [67,68]. This disparity between parental practices and evidence-based recommendations underscores the need for professional dietary guidelines that bridge cultural beliefs and scientific evidence [12], particularly within Hong Kong’s unique healthcare context.

### 4.3. Implications

This study has several implications across various domains. For ADHD researchers, the findings underscore the necessity of exploring the multifaceted nature of eating problems in children with ADHD. Further qualitative and quantitative studies are needed to capture the living experiences of families in diverse cultural and urban context, as well as develop a more comprehensive understanding of the challenges related to ADHD and eating problems. Further studies should explore the direct impact of ADHD-related impulsivity on eating behaviors to understand better the mechanisms that lead to eating disorders in this population. Longitudinal studies could track changes in eating behaviors over time, relating them to impulsivity levels and ADHD symptom management. 

While our study provides valuable insights, we acknowledge potential sampling issues. Our participant pool included 66.7% (8/12) of parents with college education or above, which may not fully represent Hong Kong’s general population. This high proportion of well-educated parents in our sample partially reflects Hong Kong’s unique socio-educational context, where there is strong emphasis on academic achievement and high educational attainment. Hong Kong’s competitive education system and cultural value placed on academic excellence may influence how parents approach their children’s ADHD and eating issues. Well-educated parents might be more proactive in seeking medical attention and participating in research studies, which could explain their overrepresentation in our sample. Future studies should consider strategies to recruit participants from more diverse educational backgrounds to better represent the full spectrum of Hong Kong families.

For ADHD experts and pediatricians, a holistic approach should be adopted to manage ADHD in children, integrating specific eating problems, parental strategies, and behavioral and medical interventions. For ADHD families, parents and other caregivers of children could refer to the strategies identified in this study (e.g., using innovative methods to make meals more appealing) to improve children’s eating habits. Moreover, creating structured and supervised eating environments might help manage the impulsive eating behaviors typically seen in children with ADHD. Integrating these strategies with general ADHD treatment could lead to more holistic care and better overall outcomes for affected children.

### 4.4. Limitations

This study has several limitations. First, the sample was limited to parents of children with ADHD in Hong Kong, predominantly representing those from higher educational backgrounds, which may limit generalizability to different cultural or socioeconomic contexts. Nevertheless, the findings may be applicable to other urban Chinese populations with similar educational and socioeconomic characteristics. Second, the study focused solely on parental perspectives and lacked input from children with ADHD themselves. Third, the high heritability of ADHD suggests that some participating parents may exhibit ADHD-related emotional characteristics, which may have led to the exaggeration of certain issues and emotions, especially in group dynamics. Finally, while our smaller focus groups (2–3 participants) differed from traditional sizes (6–8), this decision addressed the unique needs of our study population, offering better scheduling flexibility, sustained engagement, and privacy protection for ADHD parents. Through multiple small groups, we achieved thematic saturation while maintaining intimate peer discussions.

## 5. Conclusions

The findings reveal that children with ADHD face eating challenges uniquely shaped by Hong Kong’s advanced metropolitan environment. The city’s intense academic pressure, limited living spaces, and parents’ demanding work schedules—characteristics typical of this developed society—significantly impact children’s eating patterns. Parents have adapted by integrating Eastern and Western approaches, leveraging Hong Kong’s world-class healthcare infrastructure of professional networks, alternative therapies, and community programs. These findings highlight the critical role of urban-specific healthcare support in addressing ADHD-related eating challenge. Evidence-based dietary guidelines are imperative to address parental misconceptions in ADHD management.

## Figures and Tables

**Table 1:** Questions for the semi-structured interview.

**For your child, what do you consider healthy eating habits?**
Do you think your child has any eating problems? If yes, please describe the specific eating problems. If not, please describe your child’s usual eating habits.
Does your child have allergic reactions due to improper diet or certain foods?
Have your child’s eating problems caused any impact on you?
How do you view the impact of ADHD medication on your child’s eating habits?
What factors determine or influence your choices and combinations of food for your child?
For your child’s eating problems, what efforts or coping measures have you taken in caring and feeding your child? How effective are they?

**Table 2:** Demographic characteristics of the participants interviewed.

Characteristics	All Participants (n = 12)	Session 1 (n = 2)	Session 2 (n = 2)	Session 3 (n = 3)	Session 4 (n = 3)	Session 5 (n = 2)
Age, mean (SD)						
Parents	40.1 (3.7)	37.5 (0.7)	37 (2.8)	40 (3.0)	44.3 (3.2)	39.5 (3.5)
Child	6.9 (0.9)	7.5 (0.7)	6.5 (0.7)	6.3 (0.6)	7.3 (1.2)	7 (1.4)
Gender (parents), n (%)						
Male	2 (16.7)	0 (0)	0 (0)	1 (33.3)	0 (0)	1 (50)
Female	10 (83.3)	2 (100)	2 (100)	2 (66.7)	3 (100)	1 (50)
Gender (child), n (%)						
Male	10 (83.3)	2 (100)	2 (100)	2 (66.7)	3 (100)	1 (50)
Female	2 (16.7)	0 (0)	0 (0)	1 (33.3)	0 (0)	1 (50)
Level of education (parents), n (%)						
Senior high school	4 (33.3)	1 (50)	1 (50)	2 (66.7)	0 (0)	0 (0)
College or above	8 (66.7)	1 (50)	1 (50)	1 (33.3)	3 (100)	2 (100)
Career (parents), n (%)						
Professional/Semi-professional	4 (33.3)	0 (0)	1 (50)	1 (33.3)	1 (33.3)	1 (50)
Unskilled worker	1 (8.3)	1 (50)	0 (0)	0 (0)	0 (0)	0 (0)
Homemaker	5 (41.7)	0 (0)	1 (50)	1 (33.3)	2 (66.7)	1 (50)
Others	2 (16.7)	1 (50)	0 (0)	1 (33.3)	0 (0)	0 (0)
Child’s BMI, mean (SD)	14.3 (1.8)	13.7 (1.3)	14.1 (1.8)	13.7 (0.7)	16.1 (2.7)	13.2 (0.05)
Treatment for children’s ADHD, n (%)						
Medicine	3 (25)	1 (50)	0 (0)	1 (33.3)	0 (0)	1 (50)
CBT	3 (25)	0 (0)	0 (0)	1 (33.3)	2 (66.7)	0 (0)
Others	1 (8.3)	0 (0)	0 (0)	1 (33.3)	0 (0)	0 (0)

Abbreviations: SD: standard deviation; ADHD: attention deficit hyperactivity disorder; CBT: cognitive behavioral therapy; BMI: body mass index.

**Table 3:** Code structures.

Themes	Subthemes	Code Units
Challenges affecting ADHD children’s eating behaviors in Hong Kong	ADHD-specific eating behaviors	Impulsive/hyperactive/attentive eating patterns ADHD medication impact ADHD-related allergic constitution impact
	Challenges related to the local educational system	School schedule impact (e.g., morning routine, course schedule) Academic workload impact (e.g., homework culture) Teacher–student interaction impact (e.g., children’s emotional problems)
	Challenges related to the urban environment	Parents’ extended working hours Reliance on domestic helpers Ubiquitous fast-food accessibility Cramped living spaces
Parents’ coping strategies in Hong Kong	ADHD dietary strategies	General dietary adjustments (e.g., rice, sugar, yogurt) Traditional Chinese dietary approaches (e.g., Chinese dietary soup)
	Behavioral management	Planning physical activity Innovating eating approaches (e.g., incentives, tableware, cooking involvement) Regulating children’s emotion
	Hong Kong healthcare resource utilization	Dual alternative therapy options (traditional and modern approaches) Health information resources (e.g., media, seminar) Professional support networks (e.g., university research, open courses) ADHD parent peer support School–family collaboration
	Urban lifestyle adaptation	Fast-paced schedule management Home environment adjustment (e.g., family member, dining space) Home–outside eating balance Domestic helper integration

## Data Availability

The research data are available from the corresponding author upon request, with personal information redacted to ensure privacy.
